# REMEDIATION: A Break in the Clouds: Rebuilding New Orleans

**DOI:** 10.1289/ehp.118-a67

**Published:** 2010-02

**Authors:** David A. Taylor

**Affiliations:** **David A. Taylor** writes for *The Washington Post and Smithsonian* and is author of *Ginseng, the Divine Root*, about the science and subculture surrounding the medicinal plant. He teaches science writing at The Writer’s Center in Maryland

Rebuilding housing in New Orleans after Hurricane Katrina has involved a massive cleanup effort: removal of millions of tons of debris, thousands of potentially hazardous appliances, and tons of hazardous waste along with consideration of underground storage tanks, Superfund sites, and disabled drinking water systems. Now nonprofit groups involved in the rebuilding effort in the city’s hardest-hit neighborhoods, including the Lower Ninth Ward, are working to ensure new homes are built on safe sites, and their efforts are starting to pay off.

After Katrina struck in August 2005, more than half of New Orleans’ homes sat for days or weeks in up to 6 feet of floodwater. Environmental damage was severe, inside and out; one study published in the December 2006 issue of *EHP* found interior mold and endotoxin levels similar to agricultural environments. Citywide, thousands of houses were demolished. [For more information on the hurricane’s environmental health sequelae, see “In Katrina’s Wake,” *EHP* 114:A32–A39 (2006).]

The New Orleans Office of Community Development has worked with nonprofit builders to restore neighborhoods with a view to environmental health and justice. One group, the Make It Right Foundation, was established in 2007 to build homes in the city’s most impoverished neighborhoods. The foundation’s goal, says executive director Tom Darden, is 150 new energy-efficient homes built with low-toxicity materials, each priced under $150,000.

To start, Make It Right assessed the neighborhood’s environmental conditions with analyses from the U.S. Environmental Protection Agency (EPA) and the Tulane/Xavier Center for Bioenvironmental Research. Howard Mielke, a research professor at the center, had mapped the city’s soils before Katrina using a high-density sampling protocol, including collection of more than 5,000 samples across the city between 1998 and 2000. The main hazard he found, as reported in the December 2008 issue of *Environmental Geochemistry and Health*, was soil lead—up to 1,700 ppm in some locations. This so-called legacy contamination came from old lead-based paint as well as decades of residue from leaded gasoline and other sources, similar to other cities. In other neighborhoods Mielke still finds lead levels of 1,000–1,700 ppm. “Kids are playing in this stuff,” he says. [For more information on legacy lead contamination, see “Lead in Air: Adjusting to a New Standard,” p. A76 this issue.]

Make It Right settled on small-scale housing on a 14-block area where the main hazard found was high soil lead (around 200 ppm, or half the EPA remediation threshold for play areas of 400 ppm). Adopting guidelines proposed in a paper Mielke co-authored in the 15 April 2006 issue of *Environmental Science & Technology*, Make It Right and another nonprofit, Global Green, added a clean 6- to 12-inch layer of river alluvium and landscaping to remediate the soil. Although the lead and other heavy metal contaminants were well below action levels, says Darden, the foundation made a policy of doubling the recommended depth of added clean soil “to be on the safe side.”

Blocks of buildings in the part of the Lower Ninth Ward where Make It Right focused were reduced to bare concrete slabs by the spring of 2007 when they broke ground for rebuilding. The builders ground up concrete demolition waste, Darden says, and recycled it onsite into stormwater retention boxes, driveways, and stone gardens. By focusing on new home construction, Make It Right obviated the need to remediate interiors. They examined all building materials for low emissions and optimal recyclability. That included using structurally insulated panels made of wood and high-density insulation foam for less moisture absorption than gypsum-based drywall.

By mid-December 2009, the foundation had completed 16 houses, all of which have been occupied. Another 20 are under construction, and there are plans to break ground on 10 more in coming months.

Mielke says other cities should follow New Orleans’ model of soil testing and cleanup. “Every city in the country needs to be mapped really well.” Of the lead-contaminated soil he adds, “You can find it in any city today, and it has major impact on children’s health.”

